# Potential Impact of Body Mass Index on the Clinical Outcome of Papillary Thyroid Cancer After High-Dose Radioactive Iodine Therapy

**DOI:** 10.3389/fendo.2022.870530

**Published:** 2022-05-26

**Authors:** Jingjia Cao, Xiaolu Zhu, Yaru Sun, Xiao Li, Canhua Yun, Wei Zhang

**Affiliations:** Department of Nuclear Medicine, The Second Hospital, Cheeloo College of Medicine, Shandong University, Jinan, China

**Keywords:** body mass index, papillary thyroid cancer, radioactive iodine, therapy, response

## Abstract

**Context:**

Obesity has been reported as a potential risk factor for the aggressiveness of papillary thyroid cancer (PTC), but the data gathered so far are conflicting.

**Objective:**

The aim of our study was to evaluate the relationship between body mass index (BMI) and aggressiveness of PTC at the diagnosis and clinical outcome.

**Methods:**

A total of 337 patients who underwent radioactive iodine (RAI) therapy between March 2017 and May 2020 were recruited. Patients were divided into four groups: underweight (BMI<18.5 kg/m^2^), normal weight (18.5-24.9 kg/m^2^), overweight (25-29.9 kg/m^2^), and obese (BMI≥ 30 kg/m^2^). Treatment and follow-up were defined according to criteria used in the 2015 ATA guidelines.

**Results:**

This study included 337 patients with PTC (71.5% women, median age 45.21 ± 13.04 years). The mean BMI was 24.2 ± 3.1 kg/m^2^. Obese groups had a higher age than the other groups (P = 0.001). Moreover, obese patients had larger tumor sizes and higher T stage, compared to overweight, normal weight, and underweight patients (P = 0.007). After a median follow-up of 32 months, 279 patients (82.7%) had achieved an excellent response (ER) to therapy. The overall ER rates were compared between groups, and they did not differ significantly.

**Conclusions:**

We demonstrated that BMI may have an additive effect on the aggressiveness of PTC, but did not have an effect on the response to therapy after high-dose RAI therapy.

## Introduction

Papillary thyroid carcinoma (PTC), a common type of endocrine tumor, comprising approximately 90% of thyroid malignancies, has been increasing for several decades (1). To a large extent, genetic and environmental elements, as well as factors about lifestyle, are mainly responsible for this phenomenon of tumor growth. As we all known, obesity is a common public health problem all over the world and the percentage of obese adults has increased stably over the past two decades in China. Epidemiological evidence has demonstrated that higher body mass index (BMI) is slightly but significantly linked with the incidence of thyroid cancer. Moreover, these data identified that obesity may be a driving factor for the development of PTC (2, 3). Despite the existence of studies demonstrating the impact of obesity on thyroid cancer, other studies failed to confirm this association between increased BMI and aggressive features of PTC (4, 5).

Since radioactive iodide (RAI) therapy is an important adjuvant remedy for the management of PTC disease, a more important issue is represented by the question of whether or not to consider increased BMI as a risk factor of poor clinical response of high-dose RAI therapy (6). Conflicting reports have also been released on the clinical outcome in obese patients with thyroid cancer. Most studies have shown no differences in clinical outcomes of thyroid cancer after RAI therapy between obese and normal weight patients (7, 8). Very recently, Mele et al. reported that their results reflected a potential role for BMI as a predictor of early DTC relapse (9). Compared with those in Western countries, the Chinese population has a diverse lifestyle and dietary habit.

Based on these considerations, we were intrigued to analyze the clinical manifestations, pathological features, and the response to RAI therapy of a group of consecutive Chinese PTC patients in order to evaluate the relationship between BMI at the time of the therapy, aggressiveness of PTC, and clinical response during the follow up.

## Materials and Methods

### Patients and Study Design

In this prospective study, PTC patients who were received RAI therapy at the Second Hospital of Shandong University were selected sequentially from March 2017 to May 2020. All participants gave written informed consent. This study complies with the Declaration of Helsinki, and it was approved by the institutional ethics committee of the Second Hospital of Shandong University (KYLL-2018(LW)013). It was also registered with the Database for Chinese Clinical Trials (registration number: ChiCTR1800018760).

The inclusion criteria to select the subjects were: a) patients with PTC confirmed by pathology, all of whom had undergone total thyroidectomy and b) patients with PTC scheduled for RAI therapy. The exclusion criteria at the time of RAI therapy were: a) patients with positive anti-thyroglobulin antibodies (TgAb), b) patients with peak thyroid stimulating hormone (TSH) levels less than 30 μIU/mL, c) lack of BMI data, and d) incomplete follow-up data.

### Anthropometric Measurements

All patients included in the cohort were measured for height and weight without shoes and outer clothing on the day of RAI therapy. BMI was calculated as weight in kilograms divided by the square of height in meters (kg/m^2^). Based on the World Health Organization (WHO) classification, patients were divided into four groups: underweight (BMI<18.5 kg/m^2^), normal weight (18.5-24.9 kg/m^2^), overweight (25-29.9 kg/m^2^), and obese (BMI≥ 30 kg/m^2^).

The possible impact of BMI on clinical-pathological characteristics (age, gender, multifocality, Hashimoto thyroiditis, and TNM stage of tumor) and response to RAI therapy was acquired from medical records. TNM staging was defined based on the 8^th^ edition of the American Joint Committee on Cancer (AJCC) staging system.

### Therapy Protocol

All of the patients were prepared by levothyroxine (LT4) withdrawal and a low iodine diet for 4 weeks. When the goal of TSH level was higher than 30 μIU/mL, radioiodine was given. Before RAI therapy, 6 h after the administration of 37 MBq 131I, the 6-h thyroid uptake of radio-iodide was measured. Serological examinations, containing TSH, pre-ablation stimulated thyroglobulin (Ps-Tg), and anti-thyroglobulin antibody (TgAb) levels, were measured on the day of RAI treatment. The standard I-131 activity administered at the start of the study period was 3700 MBq. Three days after RAI therapy, a post-treatment whole body scan (Rx-WBS) was implemented. After RAI therapy, patients were conventionally followed-up for 3 to 6 months by neck ultrasound and serum biochemical tests (consisting of serum Tg, TgAb, and TSH). Repeated RAI therapy for persistent disease was performed at an interval of 6 months.

### Assessment of Clinical Response

Based on these data, the clinical outcomes of RAI therapy were classified as excellent response (ER), indeterminate response (IDR), biochemical incomplete response (BIR), and structural incomplete response (SIR). Specific dynamic evaluation criteria are detailed in the ATA guidelines (2015). Incomplete response (IR) included BIR and SIR.

### Statistical Analyses

The results are expressed as mean ± SD for continuous data and percentage (%) for categorical data. Each group was compared using the χ2-test or Fisher’s exact test for categorical variables or the Kruskal-Wallis test for continuous variables. The first detection of ER in patients was defined as the endpoint. Cox regression analysis was used to compare the multivariable effects on patients’ initial achievement of ER and to calculate hazard ratios (HRs) and 95% confidence intervals (CIs).

## Results

### Population Characteristics

A total of 337 patients with papillary thyroid cancer were enrolled in this study. The median age was 45.21 ± 13.04 years (range 18–77 years), and 241 (71.5%) were women. The mean BMI was 24.2 ± 3.1 kg/m^2^. Overall, 51 of the patients were classified as underweight. A total of 30.8% of the patients (N=104) were normal weight, 35.6% (N=120) were overweight, and 18.4% (N=62) were obese. In total, 16.6% of the patients (N=56) also had diabetes, while 83.4% (N=281) did not have diabetes at the time of diagnosis. The frequency of a T1 T stage was 78.6%, followed by T2 (13.2%), T3 (4.2%), and T4 (4.1%). A total of 43.6% and 49.5% of patients had N1a and N1b stage, respectively. Distant metastases confined to the lungs were identified in 10 (2.9%) patients. The clinical features and response to the treatments at the end of follow up are reported in **Table 1**.

**Table 1:** Baseline characteristics of papillary thyroid cancer (n = 337).

Parameter	N (%)
Gender	
Male	96 (28.5%)
Female	241 (71.5%)
Age at diagnosis (years)	45.21 ± 13.04
Body mass index (kg/m^2^)	
Underweight	51 (15.1%)
Normal weight	104 (30.8%)
Overweight	120 (35.7%)
Obese	62 (18.4%)
Diabetes	
Yes	56 (16.6%)
No	281 (83.4%)
PTC	
CV-PTC	302 (89.6%)
FV-PTC	20 (5.9%)
Others*	15 (4.5%)
Multifocality	
No	118 (35.1%)
Yes	219 (64.9%)
T stage	
T1	265 (78.6%)
T2	44 (13.1%)
T3	14 (4.2%)
T4	14 (4.1%)
N stage	
N0	23 (6.8%)
N1a	147 (43.6%)
N1b	167 (49.6%)
M stage	
M0	327 (97.1%)
M1	10 (2.9%)
ATA Initial Risk Stratification System	
Low	41 (12.1%)
Intermediate	198 (58.8%)
High	98 (29.1%)
The first-time response to therapy	
ER	177 (52.5%)
IDR	74 (21.9%)
IR	86 (25.6%)
At the end of clinical outcome	
ER	279 (82.7%)
IDR	26 (7.8%)
IR	32 (9.5%)
Follow-up (months)	32

CV-PTC, the classical variant of papillary thyroid cancer; FV-PTC, follicular variant of papillary thyroid cancer; Others*: tall cell variant papillary thyroid cancer, sclerosing diffuse papillary thyroid cancer.

### Associations Between BMI and Clinical Features of PTC

Patients in the obese group were older than those in the other groups (*P* = 0.001). The age in the underweight group was also significantly lower. The incidence of multifocality in the obese group was higher than that in other groups (P = 0.007). Moreover, obese patients had larger tumor sizes and higher T stage, compared to overweight, normal weight, and underweight patients (*P* = 0.007). We observed no statistical significance between the PTC subtype, lymph node metastases, distant metastases, and intermediate or high risk of recurrence (all four groups). Clinical and demographic features according to BMI are presented in **Table 2**.

**Table 2:** Demographic data and aggressive features in obese, overweight, normal weight, and underweight patients.

	Underweight (n=51)	Normal weight (n=104)	Overweight (n=120)	Obese (n=62)	Statistical value	*P* value
Age	39.65 ± 12.75	45.36 ± 11.37	43.22 ± 11.23	53.39 ± 15.42	13.502	**0.001**
<55	43 (84.3%)	83 (79.8%)	94 (78.3%)	30 (48.4%)	27.073	**0.001**
≥55	8 (15.7%)	21 (20.2%)	26 (21.7%)	32 (51.6%)		
Gender						
Male	9 (17.6%)	36 (34.6%)	31 (25.8%)	20 (32.3%)	5.707	0.127
Female	42 (82.4%)	68 (65.4%)	89 (74.2%)	42 (67.7%)		
Diabetes						
Yes	10 (18.2%)	13 (13.1%)	17 (14.2%)	16 (25.4%)	4.99	0.173
No	45 (81.8%)	86 (86.9%)	103 (85.8%)	47 (74.6%)		
Multifocality						
No	19 (37.3%)	43 (41.3%)	46 (38.3%)	10 (16.1%)	12.244	**0.007**
Yes	32 (62.7%)	61 (58.7%)	74 (61.7%)	52 (83.9%)		
Tumor size	1.51 ± 0.98	1.29 ± 0.97	1.23 ± 1.05	1.86 ± 1.55	11.51	**0.009**
T stage						
T1	40 (78.4%)	85 (81.7%)	98 (81.7%)	42 (67.7%)	23.954	**0.004**
T2	9 (17.6%)	13 (12.5%)	16 (13.3%)	6 (9.7%)		
T3	0 (0.0%)	4 (3.8%)	4 (3.3%)	6 (9.7%)		
T4	2 (3.9%)	2 (1.9%)	2 (1.7%)	8 (12.9%)		
N stage						
N0	4 (7.8%)	6 (5.8%)	10 (8.3%)	3 (4.8%)	1.760	0.940
N1a	21 (41.2%)	49 (47.1%)	51 (42.5%)	26 (41.9%)		
N1b	26 (51.0%)	49 (47.1%)	59 (49.2%)	33 (53.2%)		
M stage						
M0	51 (100.0%)	101 (97.1%)	117 (97.5%)	58 (93.5%)	4.267	0.234
M1	0 (0.0%)	3 (2.9%)	3 (2.5%)	4 (6.5%)		
HT						
No	41 (80.4%)	97 (93.3%)	103 (85.8%)	55 (88.7%)	6.012	0.111
Yes	10 (19.6%)	7 (6.7%)	17 (14.2%)	7 (11.3%)		
ATA stratification						
Low	4 (7.8%)	13 (12.5%)	14 (11.7%)	10 (16.1%)	3.916	0.688
Intermediate	34 (66.7%)	60 (57.7%)	73 (60.8%)	31 (50.0%)		
High	13 (25.5%)	31 (29.8%)	33 (27.5%)	21 (33.9%)		
131I-uptake	2.59 ± 0.32	2.64 ± 0.31	2.61 ± 0.39	2.51 ± 0.24	8.85	**0.03**
Tg level (ng/mL)						
<10	42 (82.4%)	80 (76.9%)	91 (75.8%)	37 (59.7%)	9.168	**0.027**
≥10	9 (17.6%)	24 (23.1%)	29 (24.2%)	25 (40.3%)		
TSH level						
Response to therapy						
ER	28 (54.9%)	53 (51.0%)	64 (53.3%)	32 (51.6%)	10.649	0.100
IDR	14 (27.5%)	22 (21.2%)	31 (25.8%)	7 (11.3%)		
IR	9 (17.6%)	29 (27.9%)	25 (20.8%)	23 (37.1%)		

HT, Hashimoto thyroiditis; LNM, lymph nodes mets. The bold label means statistically significant difference.

In this series of patients, a correlation between BMI and age was noted (Spearman’s value: 0.271, *P*=0.001). The relationship of BMI with other factors is outlined in **Table 3**.

**Table 3:** The relationship of BMI with other factors.

	Correlation *r*	*P* value
Sex (n)	-0.039	0.480
Age (years)	0.271	**0.001**
Multifocality (n)	0.135	**0.013**
T stage (n)	0.093	0.085
N stage (n)	0.023	0.672
M stage (n)	0.093	0.090
131I-uptake	-0.055	0.311
TSH level (mIU/L)	0.048	0.378
Tg level (ng/mL)	0.140	**0.01**

The bold label means statistically significant difference.

### Correlation Between BMI and Clinical Outcomes

**Figure 1:**
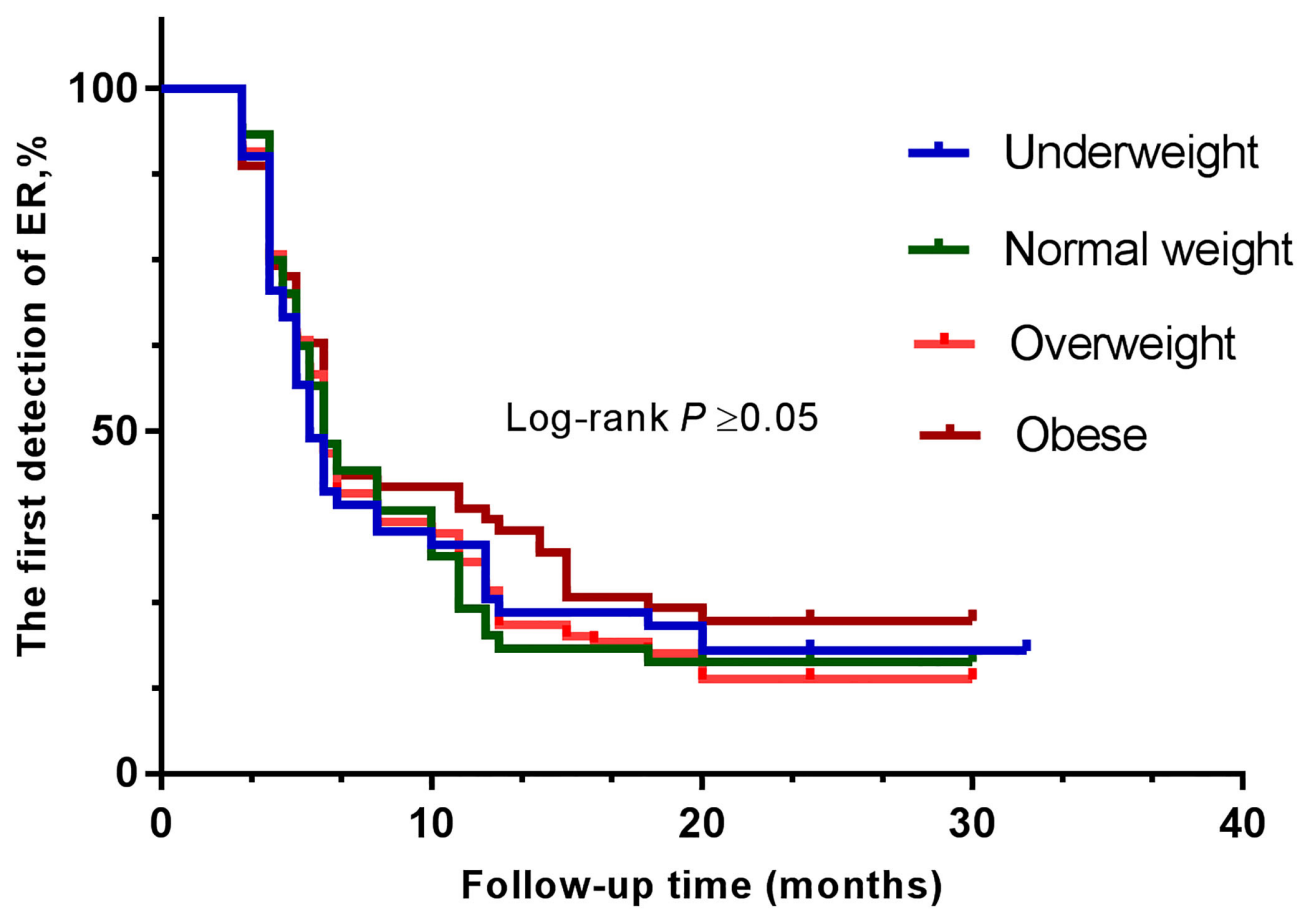
Kaplan-Meier curves show the results of the first detection of ER in patients with PTC. The patients were divided into four groups—underweight (blue line), normal weight (green line), overweight (orange line), and obese (brown line).

**Figure 2:**
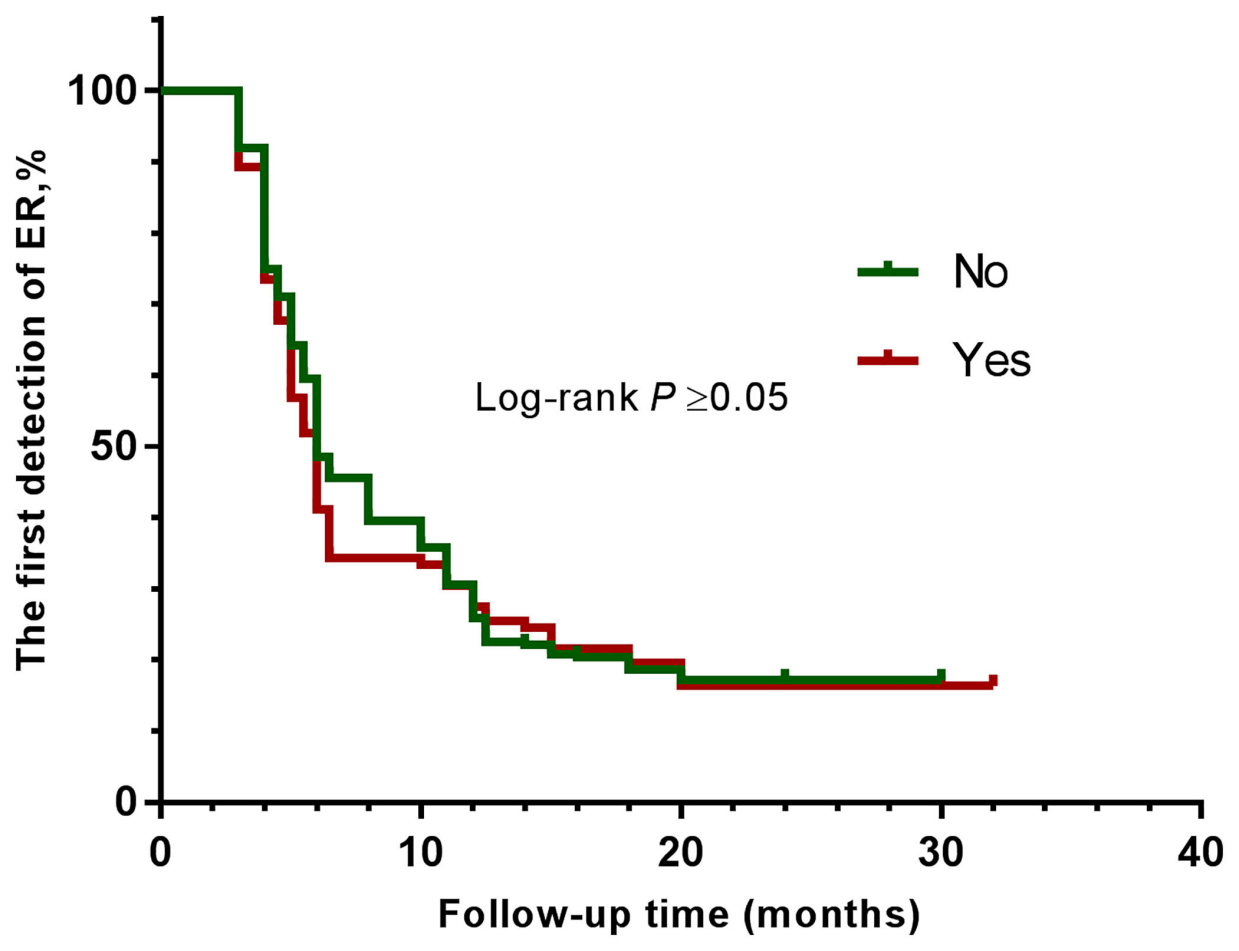
Kaplan-Meier curves show the results of the first detection of ER in patients with PTC. The patients were divided into two groups—without diabetes (green line), and with diabetes (brown line).

The clinical efficacy was assessed at the end of the study, and the median follow-up duration was 32 months. After high-dose radioactive iodine therapy, 279 patients (82.7%) achieved an excellent response. The overall ER rates were compared between groups using the log-rank test (**Figures 1**, **2** and **Table 4**). They did not differ significantly according to BMI (all four groups) or diabetes.

**Table 4:** The Cox regression analysis for the initial achievement of ER to RAI therapy.

ER/(IDR+IR)	Univariate			Multivariate		
	HR	95%CI	P value	HR	95%CI	P value
Gender						
Male	Ref.			Ref.		
Female	1.270	0.978-1.650	0.073	1.172	0.890-1.543	0.258
Age						
<55	Ref.					
≥55	0.942	0.721-1.230	0.659			
Diabetes						
Yes	Ref.					
No	1.828	0.742-2.167	0.532			
T stage						
T1	Ref.			Ref.		
T2	1.538	0.816-2.899	0.183	0.892	0.613-1.298	0.550
T3	1.161	0.572-2.357	0.671	0.673	0.331-1.370	0.275
T4	0.671	0.272-1.651	0.385	0.679	0.349-1.321	0.254
N stage						
N0	Ref.			Ref.		
N1a	0.879	0.554-1.395	0.584	0.649	0.397-1.063	0.086
N1b	0.654	0.412-1.037	0.071	0.470	0.277-0.796	0.005
M stage						
M0	Ref.			Ref.		
M1	0.044	0.005-0.415	**0.006**	0.075	0.018-0.311	**0.001**
ATA						
Low	Ref.			Ref.		
Intermediate	1.104	0.760-1.602	0.605	1.517	0.987-2.333	0.058
High	1.025	0.681-1.543	0.906	1.646	1.055-2.568	**0.028**
Tg level						
<10	Ref.					
≥10	0.344	0.253-0.467	**0.001**			
131I-uptake	0.610	0.415-0.898	**0.012**	0.681	0.437-1.059	0.088
BMI						
Underweight	Ref.			Ref.		
Normal	1.139	0.751-1.729	0.539	1.229	0.838-1.803	0.291
Overweight	1.177	0.827-1.675	0.366	1.168	0.810-1.683	0.406
Obese	0.182	0.839-1.666	0.338	1.208	0.782-1.866	0.395

The bold label means statistically significant difference.

## Discussion

Although there is no definite link between thyroid cancer and increased BMI, the suggestion that obesity is the cause of many chronic diseases and special types of cancer is widely accepted. A series of studies have researched the role of obesity in the aggressiveness of the course of thyroid cancer (10, 11). However, these results are mixed. In a cohort of 2,057 thyroid cancer patients, for example, Kim et al. demonstrated that a higher level of BMI was associated with advanced TNM stage (12). However, Matrone et al. found no connection between aggressiveness and BMI in thyroid cancer patients (13). These conflicting data may partially be explained by differences in population characteristics, sample capacity, and BMI threshold setting. We investigated the influence of BMI as a continuous variable on aggressive features and clinical responses in PTC.

We evaluated the clinical features of 337 consecutive PTC patients after high-dose radioactive iodine therapy. We did not find any significant differences regarding histology, Hashimoto thyroiditis, and N stage among the four groups of patients. However, our results are in line with those of Kim et al., in which the researchers did find that higher BMI was significantly associated with larger tumor size, multifocality, and more advanced T stage. By contrast, Kwon et al. showed that higher BMI was not related with clinical features of thyroid cancer (11). In their study, the median BMI value was 28.1 kg/m^2^. In our study, the median BMI value was lower (24.2 kg/m^2^). Typically, Asians are less obese, and their habitual diet is different to that of Caucasians. In the Kim et al. study, the median BMI was 23.8 kg/m^2^ and all patients were Korean. The population constitution of our research resembled the range of obesity and race in the Kim et al. study, which may be responsible for the similarity of results. Findings identical to those of Kim et al. were also obtained in studies performed in China (14, 15). However, Chiara Mele et al. highlighted a potential association between metabolic setting, circulating adipokines, and thyroid cancer phenotype. And it was confirmed that obesity was associated with lymph node metastases in patients with DTC (9).

A lack of connection between BMI and DTC aggressive features was also documented by Polish et al. in a large cohort of 1,181 patients (16). In that study, approximately 30% of patients did not undergo radioactive iodine therapy and about 70% of the patients were low risk. Unlike with the Polish study, all of our patients underwent total thyroidectomy, followed by radioactive iodine treatment. Furthermore, compared to the Polish study, our study group revealed aggressive features due to a greater number of intermediate and high recrudescence risk patients (58.8% vs 26.4%; 29.1% vs 18.7%).

Besides, our research has no evidence to confirm that thyroid cancer with diabetes promotes the invasive growth of tumors. However, Li et al. has shown that PTC complicated with diabetes has a higher risk of invasive tumor growth (17). In our study, the ratio of thyroid cancer with diabetes was low, which may affect the role of diabetes in tumor invasiveness and clinical outcome.

In addition, higher TSH levels have been considered as factors that boost the invasiveness of PTC (18). The positive association between TSH level and BMI has also been reported in previous studies, including a recent one that analyzed a cohort of 432 prospectively followed patients. But the serum TSH levels were similar in all four BMI groups.

As far as radioactive iodine therapy was concerned, we did observe significant differences among the four groups, in terms of 131I uptake and of the Tg level before 131I treatment. When using the definition of first reaching ER as the endpoint event, we found that no differences in the clinical outcome were noted depending on BMI. Our results agree with those of Chung et al., in which the authors did not find a significant difference between BMI groups in the outcome of the disease (19).

This work also has some limitations. Firstly, because this was a prospective study, the sample size we included may not have been large enough. Thus, this study defined obesity based solely on BMI, similar to many other studies. Thirdly, our follow-up time was shorter than other studies.

In conclusion, in our cohort of Chinese subjects we demonstrated that BMI may have an additive effect on the aggressiveness of PTC, but may not have an effect on the response to therapy after high-dose radioactive iodine therapy.

## Data Availability Statement

The raw data supporting the conclusions of this article will be made available by the authors, without undue reservation.

## Ethics Statement

The studies involving human participants were reviewed and approved by the institutional ethics committee of the Second Hospital of Shandong University. The patients/participants provided their written informed consent to participate in this study.

## Author Contributions

Conception and design: WZ. Collection and assembly of data: XZ and XL. Data analysis and interpretation: YS and CY. Manuscript writing: JC. Final approval of the manuscript: All authors.

## Conflict of Interest

The authors declare that the research was conducted in the absence of any commercial or financial relationships that could be construed as a potential conflict of interest.

## Publisher’s Note

All claims expressed in this article are solely those of the authors and do not necessarily represent those of their affiliated organizations, or those of the publisher, the editors and the reviewers. Any product that may be evaluated in this article, or claim that may be made by its manufacturer, is not guaranteed or endorsed by the publisher.
